# Recent Advances in Detergent Chemistry and Handling Support Membrane Protein Analysis Including Lipid Interactions

**DOI:** 10.1002/chem.202501549

**Published:** 2025-05-30

**Authors:** Katharina Alker, Jan‐Simon Behnke, Leonhard H. Urner

**Affiliations:** ^1^ TU Dortmund University Department of Chemistry and Chemical Biology Otto‐Hahn‐Str. 6 44227 Dortmund Germany

**Keywords:** detergent, GPCR, lipid, membrane, protein

## Abstract

Non‐ionic detergents are key reagents for the characterization of membrane protein drug targets. Suitable detergents are commonly identified by trial and error, which leads to failed preparations and raising costs. Recent findings suggest that not only the chemistry of detergents but also the strategy with which detergents and proteins are brought together impact the successes of investigations. To facilitate the future development of non‐ionic detergents in membrane protein research, herein, we review chemical design concepts and detergent exchange strategies for a successful integration of detergents into the analysis of challenging membrane protein complexes. Our overview reveals exciting opportunities to tackle existing challenges, including the stabilization of G‐protein coupled receptors, the development of fluorinated detergents for studying protein–lipid binding, top‐down design of detergents, and hybrid detergents for the identification of non‐canonical lipid associations to proteins with relevance to antibiotic research. Our review will facilitate the development of chemical tools for the biophysical characterization of membrane proteins and support the discovery of biological findings in the future.

## Graphical abstract

**Detergent design** parameters determine experimental successes in membrane protein research. Recent progresses in detergent chemistry are reviewed to promote the top‐down design of non‐ionic detergents for the purification and analysis of intact membrane proteins, including lipid interactions.

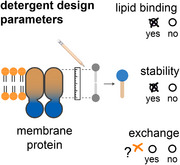

## 1 Introduction

Membrane proteins are essential components of cell membranes that play crucial roles in vital processes, such as signaling, nutrient transport, enzymatic activity, and cell adhesion.^[^ ^1^ ^]^ Membrane proteins have hydrophobic domains that are embedded in cell membranes and are difficult to isolate for analysis without perturbing their structure.^[^ ^2^ ^]^ This poses significant challenges for researchers that are attempting to study the structure of membrane proteins in life sciences and drug discovery.

**Scheme 1:**
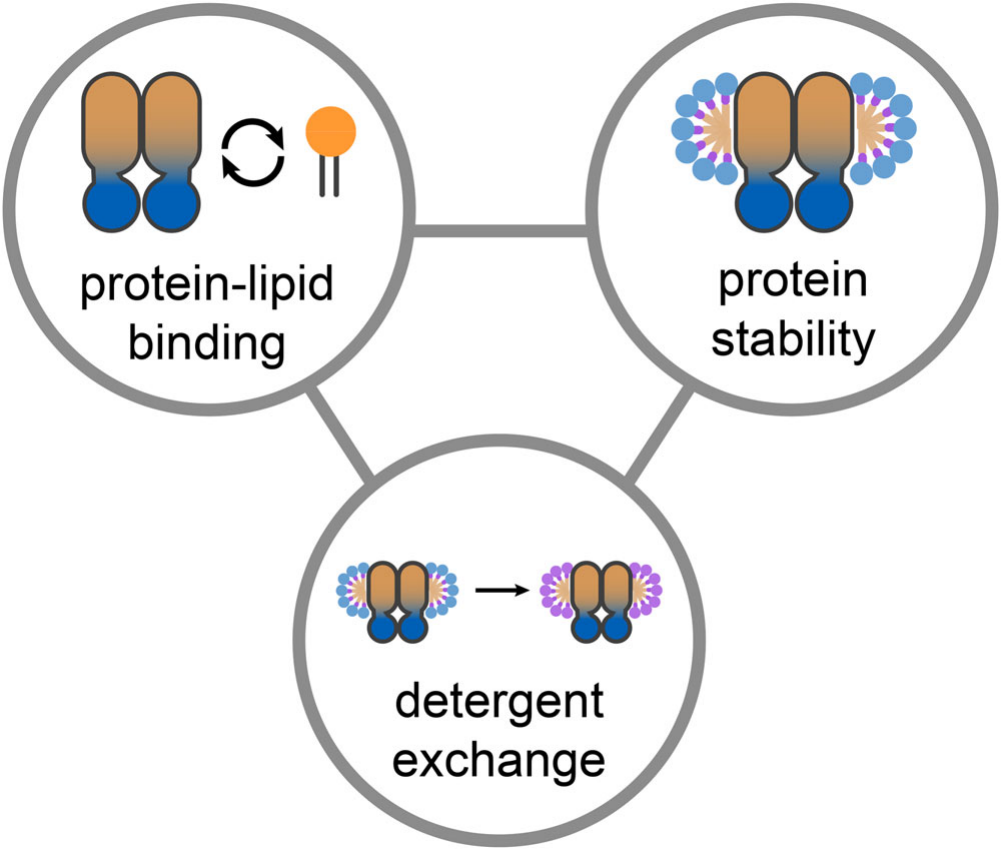
Overview of membrane protein purification parameters. Recent efforts in detergent chemistry were devoted toward the optimization of protein–lipid binding, protein stability, and detergent exchange.

Detergents are essential tools that enable the extraction of intact membrane protein complexes from cell membranes.^[^ ^3^ ^]^ Recent efforts in detergent chemistry were devoted toward the optimization of membrane protein purification parameters, including protein stability, protein–lipid binding, and detergent exchange (Scheme 1). Ideal detergents maintain protein structure and stability in solution to enable purification and structural analysis (Scheme 1). Furthermore, ideal detergents can be used to control protein–lipid interactions during purification. Co‐purifying lipids can be either identified or added back to delipidated proteins to study the role of individual lipids for protein structure and function (Scheme 1).^[^ ^4^, ^5^ ^]^ Additionally, ideal detergents are compatible with functional assays^[^ ^6^ ^]^ and biophysical measurements, such as circular dichroism spectroscopy,^[^ ^7^ ^]^ crystallography,^[^ ^8^ ^]^ nuclear magnetic resonance spectroscopy,^[^ ^9^ ^]^ and native mass spectrometry.^[^ ^10^ ^]^

The current landscape of detergents is vast and no detergent is suitable for all applications. Therefore, detergents are frequently exchanged between individual purification steps and applications, which complicates the detergent selection process and requires empirical testing. Moreover, the degree to which detergents are exchanged varies with utilized detergent exchange strategies and creates an invisible, yet, underappreciated bias on experimental outcomes. Altogether, contributes to the universal dogma that the selection of detergents depends more on empirical factors than on scientific principles.^[^ ^11^, ^12^, ^13^ ^]^

To support the future development of detergents for membrane protein purification and analysis, herein, we review related literature published since 2022. Literature reviews published before 2022 summarized how detergent chemistry can affect membrane solubilization,^[^ ^14^, ^15^ ^]^ protein–detergent interactions,^[^ ^16^, ^17^, ^18^ ^]^ protein–lipid interactions,^[^ ^19^ ^]^ alternative membrane mimetics,^[^ ^20^, ^21^ ^]^ and structure determination, i.e., crystallography,^[^ ^22^ ^]^ cryo‐electron microscopy,^[^ ^22^, ^23^ ^]^ and native mass spectrometry.^[^ ^5^, ^10^, ^24^ ^]^ Herein, we complement the repertoire of reviews by summarizing the latest progresses that have been made in detergent chemistry, including detergent design concepts for membrane protein stabilization, the investigation of protein–lipid interactions by native mass spectrometry, and detergent exchange strategies. Finally, we interrogate how recent findings in detergent chemistry can stimulate future progress in the biophysical characterization of membrane proteins and membrane biochemistry.

## 2 Results and Discussion

### 2.1 Hybrid Detergents Control Protein Delipidation

A detergent that can be universally applied for the purification and analysis of intact membrane proteins is highly desired, because it can simplify membrane protein studies and support the discovery of novel biological findings with no delay. An important aspect of being able to purify intact membrane proteins with detergents is the co‐purification of membrane lipids during extraction and affinity purification, because lipids can help to stabilize protein structure and function.^[^ ^19^, ^20^ ^]^

**Figure 1:**
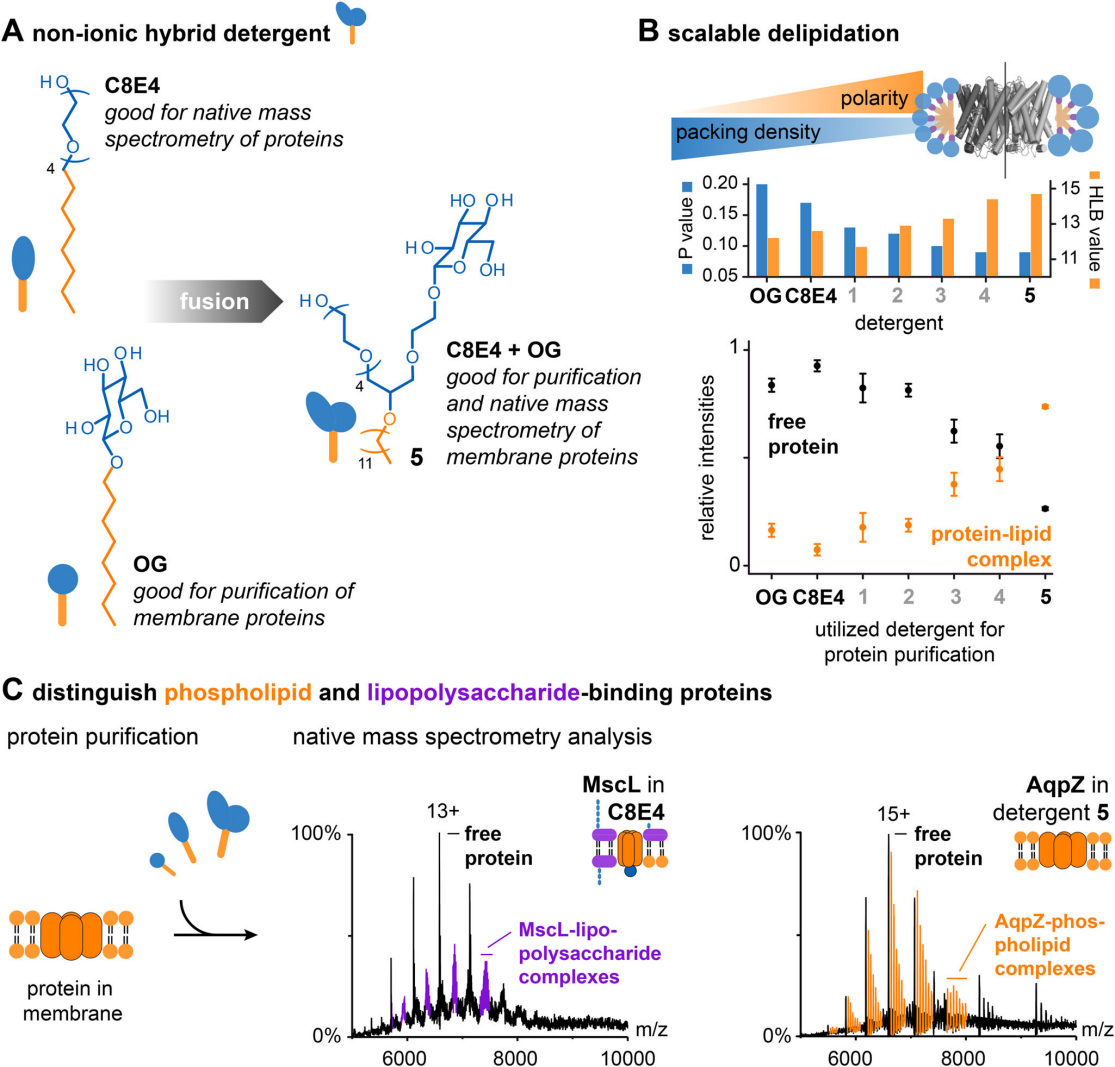
Hybrid detergents control membrane protein delipidation. A) Structure of a hybrid detergent that combines features of detergents that are good for protein purification and mass spectrometry analysis. B) Bar chart showing trends in HLB values and *p* values to visualize changes in polarity and molecular shape among different detergents. C) Mass spectra of membrane proteins obtained upon purification with different detergents can be used to distinguish PL‐ and LPS‐binding membrane proteins under the experimental conditions employed. Figure was taken from Ref. [31] with permission from Wiley Analytical Sciences.

The delipidating properties of detergents depend on the chemical nature of the detergent head group, hydrophilic–lipophilic balance (HLB), conical shape, and utilized detergent concentration.^[^ ^24^ ^]^ Native mass spectrometry is increasingly used to probe relative delipidation outcomes after protein purification.^[^ ^24^ ^]^ Most detergents either retain protein–lipid interactions during purification or enable native mass spectrometry analysis.^[^ ^5^, ^25^ ^]^ Hybrid detergents were recently highlighted due to their outstanding compatibility with protein purification and native mass spectrometry.^[^ ^26^, ^27^ ^]^ For example, the reference detergents tetraethylene glycol monooctyl ether (C8E4) and n‐octyl‐β‐D‐glucoside (OG) remove lipids almost quantitatively from proteins during purification (Figure 1A,B).^[^ ^25^, ^26^ ^]^ To obtain a hybrid detergent (C8E4 + OG) that enables both the purification and native mass spectrometry analysis of protein–lipid complexes, the polar head groups of C8E4 and OG were chemically fused (Figure 1A,B).^[^ ^26^ ^]^ This achievement can be explained by gradual changes in polarity and molecular shape that naturally occurred during the structural evolution of hybrid detergents.^[^ ^19^, ^26^, ^28^ ^]^ To better visualize changes in polarity and shape, HLB values and packing parameters (*p*) were used.^[^ ^29^, ^30^ ^]^ The lower the *p* value, the more conical the shape of a detergent (Figure 1B). The higher the HLB values, the more polar the detergents (Figure 1B). To control protein delipidation, a carefully designed protein purification pipeline was employed in which proteins were extracted from membranes and purified under comparable conditions.^[^ ^26^ ^]^ The authors assessed the delipidation status of the proteins by native mass spectrometry and found that the degree to which membrane proteins co‐purified with phospholipids (PL) increased the more polar and the more conically shaped the detergents are (Figure 1B).^[^ ^26^ ^]^ This is expected since non‐ionic detergents compete with protein–PL binding by hydrophobic interactions. More conically‐shaped, polar detergents likely form more loosely packed, polar detergent aggregates, which can facilitate the uptake of PLs into proteomicelles. In comparison to protein–PL complexes, the delipidation of membrane proteins that bind lipopolysaccharides (LPS) was not sensitive to changes in detergent polarity and shape.^[^ ^26^ ^]^ Attractive electrostatic and hydrophobic interactions between proteins and LPS are significantly strengthened by the charged oligo‐glycan structure and hydrophobic tails of LPS, thus leading to stronger protein binding compared to PLs. Important for the biological investigation of protein–lipid interactions was that PLs and LPS differ in terms of their molecular weights. Both types of protein–lipid complexes can be differentiated at a glance upon purification and native mass spectrometry analysis with hybrid detergents (Figure 1C).^[^ ^26^ ^]^

A current limitation of hybrid detergents bearing a single alkyl tail is the limited utility for protein extraction from membranes.^[^ ^19^, ^28^ ^]^ Hybrid detergents with a single alkyl tail are too hydrophilic for the disruption of lipid membranes, which is reflected in reduced protein quantities upon extraction and affinity purification.^[^ ^19^, ^28^ ^]^ Future efforts will clarify whether the installation of more than one alkyl tail, such as in the cases of glycan‐based detergents from the Chae group,^[^ ^32^ ^]^ will unlock the full potential of hybrid detergents for the direct purification of proteins from membranes.

### 2.2 HLB Enables Streamlined Detergent Optimization

**Figure 2:**
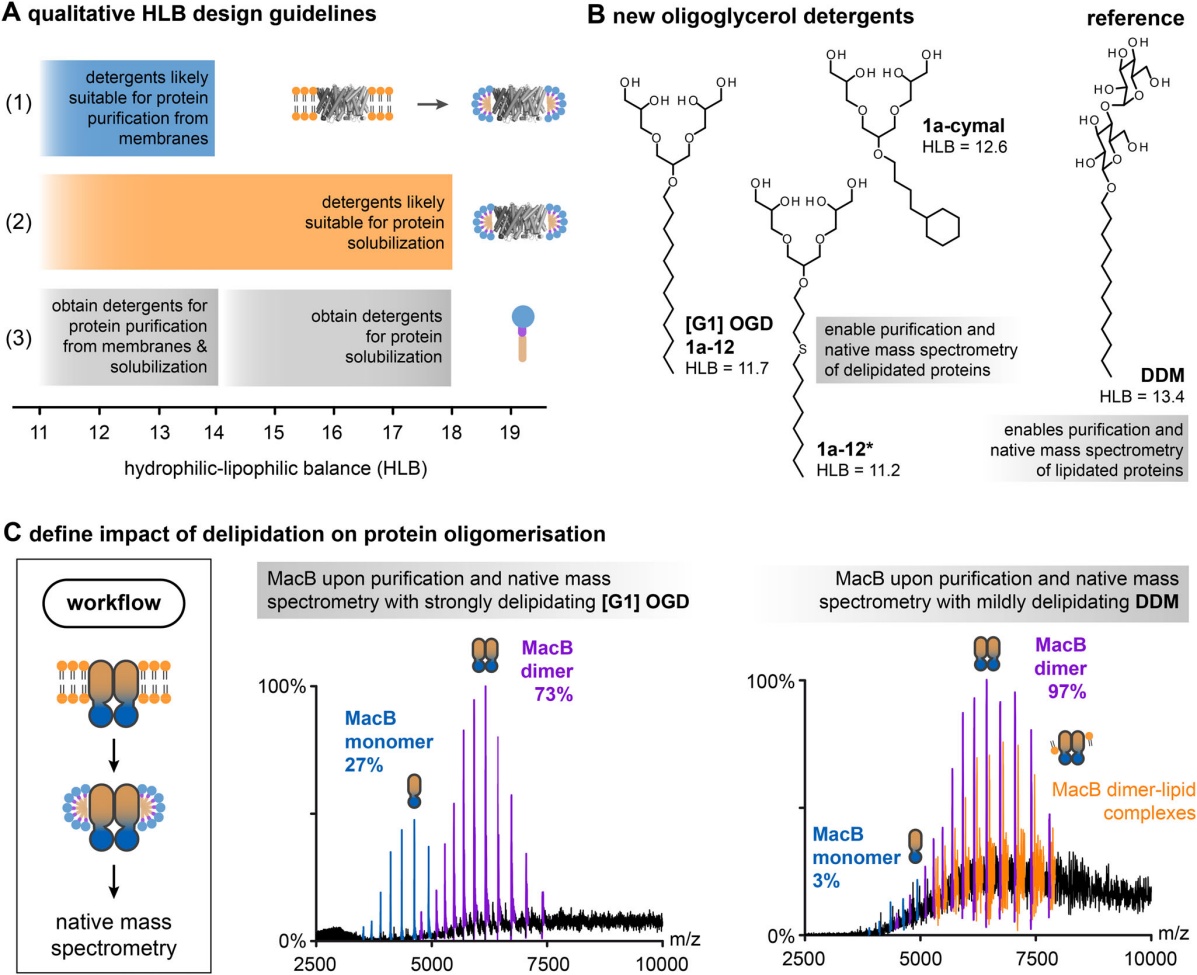
Detergent optimization for the investigation of membrane proteins. A) Qualitative HLB guidelines can be used to assess the utility of non‐ionic detergents for different aspects of protein purification. B) Molecular structures of strongly delipidating oligoglycerol detergents and the mildly delipidating reference DDM. C) Native mass spectrometry data from the oligomer equilibrium of the bacterial drug efflux pump MacB upon purification with mildly and strongly delipidating detergents.^[^ ^33^ ^]^ The image was taken from Ref. [33] and modified with the permission of the authors (CC BY 4.0).

The idea to optimize detergents for the extraction and affinity purification of proteins through modifications in the hydrophobic tail was recently brought further to deliver quantitative HLB guidelines.^[^ ^28^, ^33^ ^]^ The utility of detergents for the purification of intact membrane proteins is determined by the balance of hydrophilic and lipophilic groups.^[^ ^33^, ^34^, ^35^, ^36^ ^]^ The balance of hydrophilic and lipophilic groups is frequently estimated by Griffin's HLB values.^[^ ^30^ ^]^ Recently, HLB values were used to estimate how likely a non‐ionic detergent will be suitable for the extraction, affinity purification, and/or solubilization of proteins upon detergent exchange. An analysis of the top 20 non‐ionic detergents in membrane protein purification indicated that detergents with HLB values between 11 and 14 are likely suitable for protein extraction and affinity purification (Figure 2A).^[^ ^33^ ^]^ Detergents with HLB values between 11 and 18 are likely suitable for protein solubilization (Figure 2A).^[^ ^33^ ^]^ The authors concluded that every detergent class, as defined by the chemical structure of the headgroup, has individual optimal HLB ranges in which it is likely to find structurally alike detergent derivatives that are equally suitable for protein purification.^[^ ^33^ ^]^

The implementation of the HLB concept in detergent optimization for protein purification recently led to two new [G1] OGDs that are suitable for the extraction and affinity purification of bacterial membrane proteins, i.e., [G1] OGD 1a‐C12* and [G1] OGD 1a‐cymal (Figure 2B).^[^ ^33^ ^]^ Compared to the reference detergent DDM, all [G1] OGDs had strong delipidating properties. This led to the idea to harness the delipidating properties of detergents for understanding how lipids affect membrane protein oligomerization.^[^ ^33^ ^]^ For example, the drug efflux pump MacB was purified under comparable conditions in strongly delipidating [G1] OGD 1a‐C12 and mildly delipidating DDM. Subsequent native mass spectrometry experiments revealed that the monomer–dimer equilibrium of MacB got shifted toward lipidated dimers when the detergent environment was switched from [G1] OGD 1a‐C12 to DDM (Figure 2C). Control re‐lipidation experiments on MacB in [G1] OGD 1a‐C12 confirmed this observation, which underlined the importance of standard *E. coli* phospholipid classes (PE, PG, CDL) for stabilizing the functional dimer of MacB.^[^ ^33^ ^]^ This knowledge delivers a blue‐print for the design of reconstructed lipid environments that resemble native lipid environments as close as possible to support the translation of findings from in vitro models into in vivo systems.^[^ ^5^ ^]^ Qualitative HLB guidelines will facilitate the assessment and optimization of detergents for the purification of membrane proteins, including their complexes with lipids. To improve the link between HLB model and detergent structure, a chromatographic method was established to specify the role of the detergent linkers (thioether, ether, triazole, amide) in functioning as an extension of the polar head and/or non‐polar tail.^[^ ^37^ ^]^

### 2.3 Detergent Design for Enhanced Protein Stabilization

#### 2.3.1 Glycan Detergents

Having reviewed recent improvements in the discovery of detergents for the extraction, affinity purification, delipidation, and native mass spectrometry analysis of membrane proteins, we now focus on recent developments in the design of detergents for membrane protein stabilization. In structural studies, such as in the cases of native mass spectrometry, crystallography, or cryo‐EM, it can be beneficial to reduce the conformational flexibility of membrane proteins during purification. This is particularly relevant for mammalian proteins, which exhibit a greater conformational flexibility than bacterial membrane proteins.^[^ ^38^, ^39^ ^]^ In membranes, conformational flexibility of protein can be reduced by lateral membrane pressure. The absence of lateral pressure, once the 2D structure of membranes is solubilized by detergents, can lead to an increase in the conformational flexibility of proteins and promote protein unfolding. Strategies to reduce conformational flexibility during protein purification are crucially needed to facilitate the investigation of medically relevant drug targets, including ion channels, solute carriers, and G‐protein‐coupled receptors (GPCRs). 

One way to overcome this obstacle is the conformation‐selective purification of membrane proteins. For example, Agasid and coworkers could stabilize a class B GPCR in a flexible detergent, such as [G1] OGD 1a‐C12, by supplementing purification buffers with a ligand that stabilizes a certain receptor conformation.^[^ ^40^ ^]^ Lipids can also be added back to micelles as conformation‐stabilizing ligands.^[^ ^38^ ^]^ Alternatively, to prevent protein unfolding in the absence of lateral membrane pressure, the Chae group follows the idea to reduce the conformational flexibility of proteins by enhancing detergent–detergent interactions. Chemically, various design approaches have been leveraged to accomplish this aim, including the balance of rigid and flexible detergent building blocks,^[^ ^41^ ^]^ synthetic accessibility,^[^ ^42^ ^]^ alkyl chain density in micelles,^[^ ^43^ ^]^ hydrophobicity,^[^ ^44^ ^]^ dynamic water‐mediated hydrogen‐bond networks,^[^ ^45^ ^]^ foldability,^[^ ^46^ ^]^ unsymmetry,^[^ ^47^, ^48^ ^]^ peptide scaffolded detergents,^[^ ^49^ ^]^ fluorinated detergents with a lactobionamide head,^[^ ^50^ ^]^ zwitterionic head with fluorinated chains,^[^ ^51^ ^]^ rigidity,^[^ ^52^, ^53^ ^]^ surfmers, ^[^ ^54^ ^]^ and nanodiscs.^[^ ^55^ ^]^

**Figure 3:**
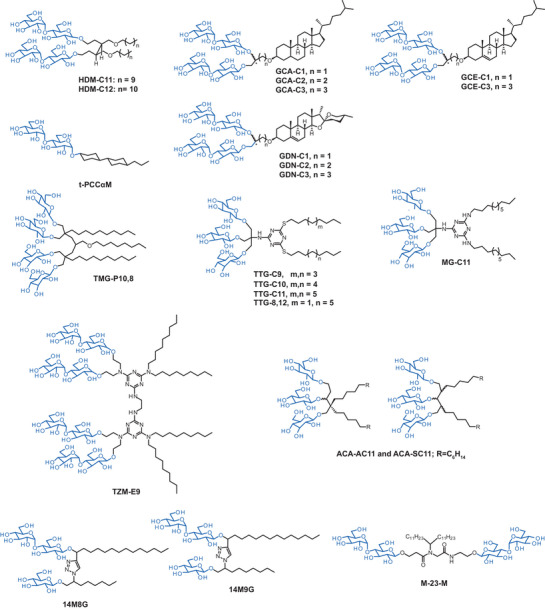
Glycan detergent design for enhanced protein stabilization. An overview of inherent structural features of detergents synthesized and analyzed aiming to increase protein stability. All detergents contain glycan‐based, polar head groups (blue) and different hydrophobic parts or linker.

To reduce protein aggregation of eukaryotic membrane protein complexes, a novel class of 3,4‐bis(hydroxymethyl)‐hexane‐1,6‐diol‐based maltosides (HDMs) was prepared to leverage the concept of balancing rigid and flexible groups.^[^ ^41^ ^]^ The trick in the design of HDMs was that the rigid neopentyl glycol linker in the reference detergent lauryl maltose neopentyl glycol (LMNG) was replaced by a flexible hexane‐1,6‐diol unit (Figure 3). Interestingly, both flexible HDM detergents stabilized certain GPCRs in solution more efficiently than the more rigid reference LMNG, which highlighted the utility of tuning detergent rigidity for stabilizing membrane proteins.

Another class of rigid detergents that is frequently used in membrane protein research are glyco‐diosgenins (GDN).^[^ ^42^ ^]^ Their synthesis is tedious, which limits the widespread use in protein purification and analysis. To overcome this shortcoming, new glyco‐steroid‐based detergents were developed containing a branched dimaltoside head group, different linkers that are connecting maltose to hydrophobic groups with varying head‐to‐tail distances (C1, C2, C3, shown in Figure 3).^[^ ^42^ ^]^ Three sets of detergents (GCAs, GCEs, GDNs) were synthesized using cholestanol, cholesterol, and diosgenin, which differed in ring planarity and hydrophobicity. Although these new detergents could not extract MelB from membranes as efficient as reference detergents, they were easier to synthesize than previous analogues and some of the new detergents outperformed DDM and the original GDN detergents in stabilizing GPCRs.

Increasing the rigidity of the detergent structure was also a key strategy in the design of 4‐trans‐(4‐trans‐propylcyclohexyl)‐cyclohexyl α‐maltoside (t‐PCCαM).^[^ ^53^ ^]^ The incorporation of cyclohexyl rings into the hydrophobic tail increased the rigidity of the molecule compared to the linear alkyl chain in DDM. t‐PCCαM formed micelles with a less flexible hydrophobic core, which led to more compact protein–detergent complexes. This characteristic is beneficial for structural studies, as it can improve crystallization and cryo‐EM analysis of membrane proteins.^[^ ^53^ ^]^

Another approach to improve protein stability in micelles is to strengthen detergent–detergent interactions by leveraging the hydrophobic effect. Following this principle, new variants of tandem malonate‐derived glucosides (TMGs) were developed with a focus on increasing the alkyl chain density within micelles (Figure 3).^[^ ^43^ ^]^ Following this aim, TMGs were decorated with three alkyl chains. One candidate, i.e., TMG‐P10,8, was notably effective in extracting and stabilizing membrane proteins and outperformed the previous TMGs that featured only two alkyl chains (Figure 3).

To improve the long‐term stability of membrane proteins in detergents, tris‐(hydroxymethyl)aminomethane linker‐bearing triazine‐based triglucosides (TTGs) were developed (Figure 3).^[^ ^44^ ^]^ The TTG detergents consisted of a 1,3,5‐triazine core that was connected to two alkyl chains and a headgroup, which contained three glucose units (Figure 3). The study evaluated TTGs against conventional detergents and identified three TTG detergents that were better or at least comparable to LMNG.^[^ ^56^ ^]^ The authors proposed that the hydrophobicity of the thioether linkage, the removal of *cis/trans* isomerism associated with an amide linkage, and an optimal HLB of TTG detergents improved protein stability.^[^ ^44^ ^]^ A complementary approach to strengthen detergent–detergent interactions is to substitute symmetric alkyl tails in TTG detergents with unsymmetric alkyl tails.^[^ ^47^ ^]^ This concept led to the detergent TTG‐8,12, which outperformed symmetric analogues and LMNG in stabilizing GPCRs, like β_2_AR and MOR (Figure 3).^[^ ^47^ ^]^

Complementary to the hydrophobic effect, hydrogen bonding can be leveraged to enhance protein stabilization in the environment of a detergent micelle. Following this idea, a new class of melamine‐cored glucosides (MGs) was developed, whose melamine core contained multiple hydrogen bond donor and acceptor groups (Figure 3).^[^ ^45^ ^]^ The alkyl chains were linked to the melamine core via an amine linkage and the glucose headgroups were linked to the core through a tris(hydroxymethyl)‐aminomethane linker (Figure 3). Molecular dynamics simulations suggested that the detergent MG‐C11 formed dynamic, water‐mediated hydrogen‐bond networks within proteomicelles that enhanced detergent–detergent interactions and led to outstanding membrane protein stabilization (Figure 3).^[^ ^45^ ^]^

To further improve the idea of leveraging hydrogen bonding to enhance protein stabilization in detergent micelles, tandem triazine‐based maltosides (TZMs) were developed that exhibited the feature of foldability.^[^ ^46^ ^]^ TZMs contained two amphiphilic triazine units that were connected by diamine linkers, specifically hydrazine (TZM‐Hs), and 1,2‐ethylenediamine (TZM‐Es) (Figure 3). Compared to more rigid TZM‐H detergents, foldable TZM‐E detergents (E8, E9, E10) could stabilize a broad range of membrane proteins. The flexibility of the TZM‐E detergent core enables the detergent to adopt folded conformations in micellar environments, which can increase the density of alkyl chains within micelles to enhance protein stability.

In analogy to the idea behind symmetric and unsymmetric maltoside detergents, 1,3‐acetonedicarboxylate‐derived amphiphiles (ACAs) were designed (Figure 3).^[^ ^48^ ^]^ Asymmetrically alkylated ACA detergents (ACA‐A) and symmetrically alkylated ACA detergents (ACA‐S) were developed (Figure 3).^[^ ^48^ ^]^ Both C11 variants of ACA‐A and ACA‐S were effective at stabilizing membrane proteins. ACA‐AC11 and ACA‐SC11 were particularly effective in maintaining the functional state of proteins like MelB and GPCRs.

In addition to more rational chemical design approaches that leverage the hydrophobic effect, hydrogen bonding networks, and rigidity, glycan‐based detergents can also be optimized empirically. A core strategy to facilitate the empirical optimization of detergents for protein stability is to simplify the synthesis of diverse chemical spaces within the detergentome.^[^ ^57^ ^]^ To expand the chemical diversity of glycan‐based detergents, a library of dimeric detergents was developed. A library of 1,2,3‐triazole pre‐assembled detergents (T‐PADs) was constructed using a combinatorial approach.^[^ ^58^ ^]^ This involved functionalized monomeric detergents and click chemistry‐mediated cross‐coupling. Accordingly, two new hybrid detergents 14M8G and 14M9G were found, which enabled high‐quality studies of the transporter MsbA and the GPCR A_2A_AR using electron microscopy and nuclear magnetic resonance spectroscopy techniques. The derivatives 14M8G and 14M9G demonstrated enhanced performance in terms of preserving long‐term stability and protein activity compared to LMNG and β‐D‐undecyl maltoside.

Another approach to expand the diversity of glycan‐based detergents with preassembled detergents was a Ugi reaction‐mediated strategy.^[^ ^59^ ^]^ Ugi is a four‐component reaction involving a ketone or aldehyde, an amine, and a carboxylic acid, which form a bis‐amide once an isocyanide is added.^[^ ^57^ ^]^ By varying the hydrophilic and hydrophobic components, the authors could optimize the HLB of detergents to improve membrane protein stability. The authors synthesized two classes of unsymmetric preassembled detergents and identified M‐23‐M as a starting point for the improved nuclear magnetic resonance spectroscopy analysis of the class B GPCR GLP‐1R (Figure 3).

#### 2.3.2 Peptide‐ and Fluorine‐Based Detergents, Surfmers, and Nanodiscs

**Figure 4:**
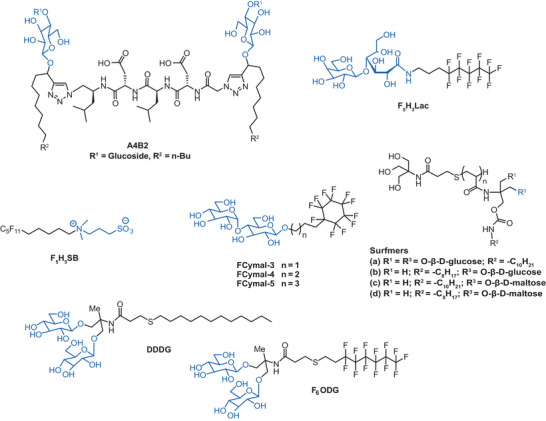
Peptide‐ and fluorine‐based detergents, surfmers, and nanodiscs for enhanced protein stability. An overview of inherent structural features of detergents that were designed to increase membrane protein stability. The polar parts of the structures are highlighted in blue.

Having reviewed the recent diversity of oligoglycerol and glycan‐based detergents, it becomes apparent that conventional detergents typically consist of polar headgroups and non‐polar hydrocarbon tail groups that can be connected by a variety of chemical linkers. Complementary, peptide‐based detergents have emerged as a unique class of membrane mimetics. Specifically, β‐sheet‐forming peptides can form well‐defined supramolecular assemblies with facial amphiphilic properties. To harness these properties for membrane protein stability, peptide‐scaffolded detergents were designed, which are hybrid molecules that are formed by preassembling detergent monomers with peptides using Click chemistry.^[^ ^49^ ^]^ Among these, the detergent variant A4B2 demonstrated superior thermal stabilization and solubilization efficiency for membrane proteins, including GPCRs. A4B2 has a low cmc and forms small micelles, which makes it particularly effective for electron microscopy studies (Figure 4).

Another modality to design detergents for membrane protein purification and analysis is to substitute hydrogen atoms by fluorine. Detergents with fluorinated hydrocarbon tails interfere less with protein–lipid interactions compared to detergents with hydrocarbon tails, which can improve the stabilization of functional proteins.^[^ ^57^ ^]^ As a result, fluorinated carbon tails can better preserve enzymatic activity.^[^ ^50^ ^]^ Fluorinated detergents exhibit hydrophobic and lipophobic properties. This means they can repel both water and lipids, which contributes to their non‐cytolytic behavior and weak interactions with the hydrocarbon chains of natural lipids.^[^ ^51^ ^]^ Finding the optimal balance between polar headgroup, hydrocarbon backbone, and degree of fluorination is not trivial and typically determined empirically. A recent case study reported lactobionamide detergents with partially fluorinated hydrocarbon chains.^[^ ^50^ ^]^ Extending the perfluorinated segment from four to seven carbon atoms reduced the cmc and water solubility. The optimal balance in polarity, hydrophobicity, and fluorophilicity for solubilizing and stabilizing the GPCR A_2A_R and ABC transporter BmrA was provided by F_5_H_3_Lac (Figure 4).

Furthermore, an improved sulfobetaine detergent F_5_H_5_SB was found to enhance protein stability and consisted of a sulfonate, quaternary ammonium group, perfluoropentyl chain, and hydrocarbon spacer (Figure 4).^[^ ^51^ ^]^

Substituting fluorine for hydrogen atoms in alkyl chains can also increase the rigidity of aliphatic tails, which can result in smaller micelles with less denaturing properties that can be more suitable for protein stabilization than hydrocarbon analogues.^[^ ^52^ ^]^ FCymal detergents consists of a polar β‐maltoside head and a perfluorinated cyclohexyl tail. The hydrogenated spacer varied from 1‐3 carbon atoms (FCymal‐3, FCymal‐4, FCymal‐5). Longer spacers, such as in the case of FCymal‐5, enhanced the stability of membrane proteins, compared to analogues with shorter spacers. Shorter spacers led to faster solubilization of lipid membranes, as seen with FCymal‐3, which solubilized lipid membranes more rapidly than FCymal‐5.(Figure 4).^[^ ^52^ ^]^

In addition to detergents, amphiphilic polymers are increasingly used as membrane mimetics that can stabilize protein structures and protein–lipid interactions.^[^ ^5^ ^]^ Polymers are macromolecules, and detergents are small molecules. This difference makes it difficult to systematically compare polymers and detergents in a structure–property study. To bridge this gap, four acrylamide‐based monomers, termed surfmers, with a glycan‐based polar head and a non‐polar alkyl chain were introduced.^[^ ^54^ ^]^ This design allows surfmers to behave like classical detergents with strong surface activity in their monomeric form. Upon polymerization, surfmers form non‐ionic amphiphilic polymers that can offer a more stabilizing environment for membrane proteins. Surfmers were more effective in solubilizing proteins from membranes, while polymers were better at maintaining the receptor's functional fold. This demonstrated the ambivalent utilities of surfmers and their polymerized counterparts in solubilizing and stabilizing fragile membrane proteins (Figure 4).^[^ ^54^ ^]^

Another membrane mimetic are nanodiscs. Nanodiscs are nanoscale, disc‐shaped lipid bilayers that are stabilized by amphiphilic molecules that shield the edges of lipid patches from water.^[^ ^55^ ^]^ Nanodiscs can mimic lipid bilayer environments surrounding membrane proteins better than detergents.^[^ ^55^ ^]^ Two amphiphiles, i.e., DDDG and F_6_ODG, were recently tested on their ability to develop small‐molecule agents that directly assemble lipids and membrane proteins into nanodiscs without the use of membrane scaffold proteins (MSPs) (Figure 4).^[^ ^55^ ^]^ F_6_ODG was specifically tailored to act in a milder way on lipid packing due to its fluorocarbon chain, resulting in gentler interactions with the lipid bilayer.^[^ ^55^ ^]^ The hydrocarbon amphiphile DDDG was able to extract the bacterial membrane protein outer‐membrane phospholipase A from phosphocholine vesicles.^[^ ^55^ ^]^ DDDG and F_6_ODG extracted a broad range of membrane proteins directly from native *E. coli* membranes. ^[^ ^55^, ^60^, ^61^ ^]^ For downstream‐applications, nanodiscs can be analyzed with Fluorescent Universal Lipid Labeling for Microfluidic Diffusional Sizing, which is a new method for the size analysis of synthetic lipid‐bilayer nanodiscs, and more complex membrane systems, such as native nanodiscs derived from cell extracts.^[^ ^62^ ^]^ Accordingly, nanodiscs are valuable tools for the biochemical and biophysical analysis of membrane proteins.

### 2.4 Detergent Exchange and Cleavable Detergents

**Figure 5:**
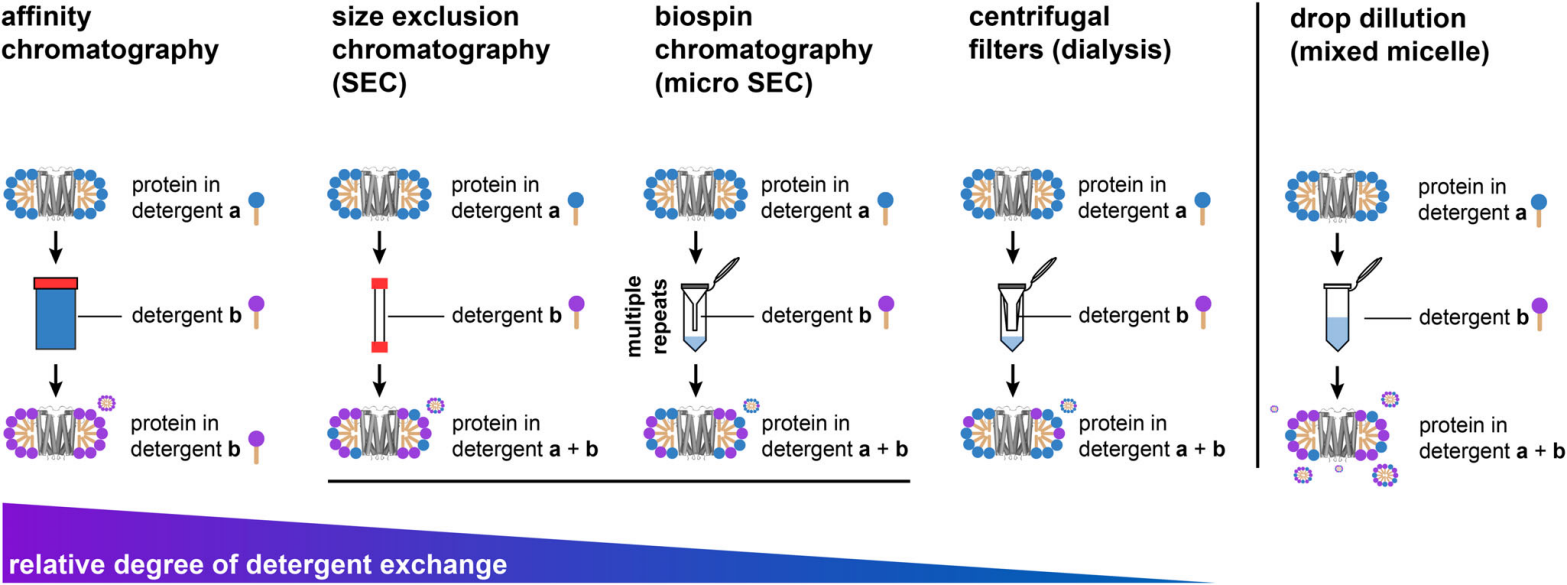
Detergent exchange strategies in membrane protein purification. Schematic visualizing different purification techniques that can be used to transfer membrane proteins from one detergent environment to another, including qualitative comparison in relative degree of detergent exchange.

Detergents that enable both the purification and analysis of intact proteins are rare. Therefore, detergents are routinely exchanged between individual steps of purification and/or analysis, for example, by affinity purification, size exclusion chromatography (SEC), biospin, or drop dilution (Figure 5).^[^ ^63^, ^64^, ^65^, ^66^, ^67^, ^68^ ^]^ A common limitation of detergent exchange strategies is that the detergent exchange is often incomplete.^[^ ^63^, ^67^ ^]^ Furthermore, if detergent quantification is not integrated in experimental pipelines, the degree to which detergents are exchanged remains unknown and creates an invisible, yet, underappreciated experimental bias. To support future membrane protein research projects, herein, we also review recent knowledge gains on the quality of detergent exchange strategies, including drop dilution, biospin, SEC, and affinity purification (Figure 5).

Leveske and coworkers provided the most recent update on the efficiency of detergent exchange strategies.^[^ ^63^ ^]^ The authors concluded that SEC can enable a more quantitative detergent exchange compared to dialysis and biospin (Figure 5). This is expected since biospin columns follow the same separation principle as SEC but have smaller column volumes and shorter column pathlengths, thus limiting the degree of detergent separation and exchange. Detergent exchange by dialysis through a semi‐permeable membrane is commonly incomplete and determined by dissociation constants of protein–detergent complexes, the maximum monomer concentration, i.e., cmc, and the molecular weights of free micelles under the employed conditions. Leveske and coworkers proposed that the cmc is the most predictive property in detergent exchange.^[^ ^63^ ^]^ The relative degree of detergent exchange was best whenever membrane proteins were transferred from detergents with low cmc values (< 1 mM) into detergents with high cmc values, such as C8E4 (∼ 8 mM) or OG (cmc ∼ 20 mM), by means of SEC or multiple rounds of biospin exchange (Figure 5). Another detergent exchange strategy is to dilute a smaller volume of a solution containing a membrane protein that was purified in a reference detergent into a larger volume of a solution containing a new detergent (Figure 5). Protein samples are typically diluted 1:10 (v:v), which leads to the formation of mixed detergent micelles (Figure 5). Even though the initial detergent is still present in the solution, this way of testing detergent exchange remains a practical and efficient approach to evaluate the utility of detergents for stabilizing membrane proteins.^[^ ^43^, ^49^, ^65^ ^]^ The most quantitative detergent exchange strategy is based on affinity purification (Figure 5). Herein, proteomicelles are immobilized of affinity resin and washed with approximately 10 column volumes of wash buffer containing the new detergent.^[^ ^69^ ^]^ In affinity purification, proteomicelles are in equilibrium with surrounding detergents. By adding fresh, detergent‐containing wash buffer, the amount of new detergent entering this equilibrium is constantly renewed, and the amount of initial detergent that was bound to the proteomicelle is constantly removed, which yields better exchange rates compared to SEC, biospin, dialysis, or drop dilution (Figure 5). A systematic comparison of the five abovementioned strategies would help to clarify the utility of these techniques for providing a better understanding of the underappreciated bias that detergent mixtures can have on downstream applications in membrane protein purification and analysis.

**Figure 6:**
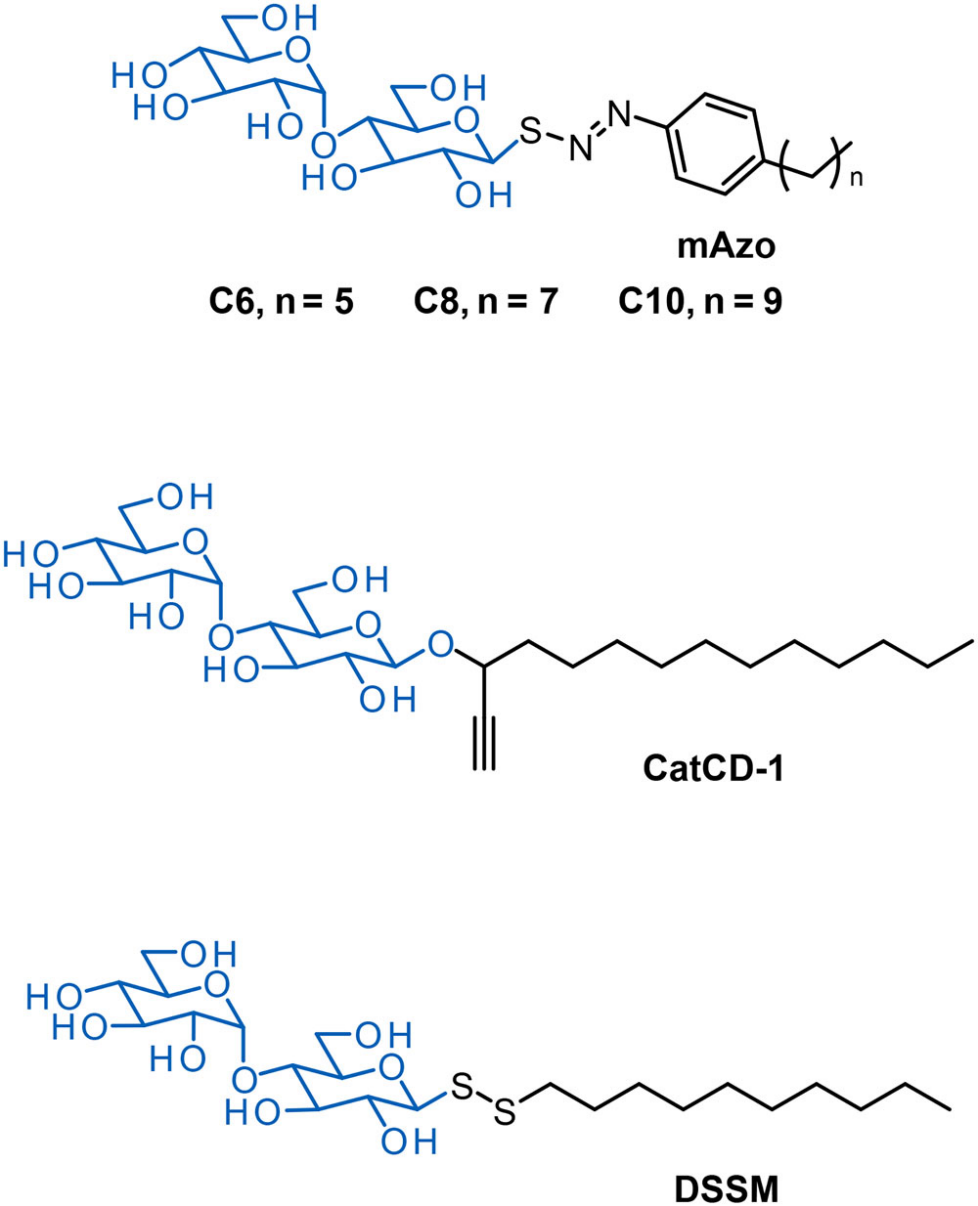
Cleavable detergents for detergent exchange. An overview of inherent structural features of detergents that were synthesized to improve detergent exchange. The detergent structures are cleavable by light (mAzo), metal catalysts (CatCD‐1) or reducing agents (DSSM). The hydrophilic parts are highlighted in blue.

A complementary approach for optimizing detergent exchange is to use cleavable detergents. Catalytically cleavable detergents (CatCDs) can be efficiently removed to facilitate a complete detergent exchange (Figure 6).^[^ ^70^ ^]^ Six new detergents (CatCD‐1 to CatCD‐6) were developed, which can be cleaved by palladium‐catalysed hydrogenolysis. CatCD‐1 demonstrated exceptional cleavage efficiency with palladium catalysts like [Pd(allyl)Cl]_2_ by cleaving more than 95% of CatCD‐1 within 2 hours in PBS buffer. After the cleavage, MsbA was successfully transferred into test detergents (LMNG, DDM, UDM, and DM (n‐decyl β‐maltoside)) without non‐specific aggregation. MsbA maintained good monodispersity, particularly in maltoside detergents. CatCD‐1 showed excellent thermal stabilization of MsbA comparable to LMNG.

To investigate the potential of photocleavable surfactants in biological applications, a new family of maltose‐derived non‐ionic surfactants containing a photocleavable azo‐sulfide linker was synthesized, which is also known as mAzo (Figure 6).^[^ ^71^ ^]^ Upon irradiation with ultraviolet light, the azo‐sulfide surfactant undergoes homolytic cleavage, resulting in the expulsion of nitrogen gas and the formation of alkylbenzene, phenolic‐derived products, and thiomaltose. Brown and coworkers tested the mAzo surfactant for membrane protein extraction. The mAzo detergent could solubilize proteins comparable to DDM, which highlighted its potential for protein purification and crystallography.

In addition to protein purification and structural analysis, mass spectrometry‐based applications can profit from the use of cleavable detergents. Detergents can interfere with mass spectrometry by hampering ionization processes or producing data artifacts that overlap with signals of species of interest.^[^ ^24^ ^]^ Cleavable detergents can be used to overcome this limitation since the initial detergent structure can be degraded on demand by using a compatible stimulus, e.g., a reducing agent or light. For example, N‐decyl‐disulfide‐β‐D‐maltoside (DSSM) mimics the properties of DDM, but includes a cleavable disulfide bond (Figure 6).^[^ ^72^ ^]^ DSSM cleavage is facilitated by the use of a reducing agent, i.e., tris(2‐carboxyethyl)phosphine, which breaks the disulfide bond and enables the removal of the detergent before or during mass spectrometry analysis. The compatibility of DSSM's with electrospray ionization mass spectrometry and reversed‐phase liquid chromatography mass spectrometry circumvents ion signal suppression, which is a common issue with traditional surfactants, like DDM. DSSM facilitated the top‐down proteomics analysis of membrane proteins, such as in the cases of the ion channel KcsA and bacteriorhodopsin. Taken together, in‐depth knowledge on the quantitative nature of detergent exchange strategies and cleavable detergents provide new opportunities for the purification and structural investigation of membrane proteins with mass spectrometry techniques.

## 3 Conclusion

In the past 4 years, we have seen a remarkable increase in chemical entities entering the detergentome to optimize the delipidation, stabilization, and sample performance for the biophysical characterization of membrane proteins. From the perspective of detergent chemistry, we can still say that there is currently no such thing as a standard detergent design approach that optimizes protein stability and sample performance for the biophysical characterization of all membrane protein systems. This is expected, because strategies for the chemical optimization of detergents depend on the intended application, the chemical nature of the detergent, and the protein. Nevertheless, the repertoire of strategies to optimize detergents has been increased remarkably and offers new, exciting opportunities to tackle existing challenges in membrane protein research.

The absence of lateral membrane pressure upon extraction into detergent micelles limits protein stability and is a common bottleneck for downstream applications.^[^ ^42^ ^]^ A powerful strategy to better mimic lateral pressure of membranes is to strengthen detergent–detergent interactions in micelles. From a chemical perspective, detergent–detergent interactions can be strengthened by leveraging the hydrophobic effect and hydrogen bonds. Both driving forces can be upregulated in detergent micelles, for example, by (i) inserting a melamine‐core into detergents that promote dynamic water‐mediated hydrogen‐bond networks,^[^ ^45^ ^]^ (ii) by increasing the alkyl tail density within micelles,^[^ ^43^ ^]^ or (iii) by introducing flexible linkers that promote foldability of detergents.^[^ ^46^ ^]^ (iv) To further improve the compatibility of protein‐stabilizing micelles for biophysical measurements, the rigidity of micellar detergent cores and/or detergent tail asymmetries can be increased.^[^ ^47^, ^48^, ^52^, ^53^ ^]^ Rigid detergent tails, such as cholesterol‐like structures, can lead to smaller micelles, which correlates to enhanced protein stability and lower interference with biophysical measurements.^[^ ^42^ ^]^ The utilities of the abovementioned strategies were mainly confirmed from studies with glycan detergents. Future studies will clarify as to whether the design concepts (i)–(iv) are transferable to the wider detergentome, which could unlock new possibilities for the purification and analysis of challenging membrane proteins.

Complementary strategies outline promising future pathways related to peptide‐ and fluorine‐based detergents. Peptide‐based detergents form amphipathic β‐sheet structures. These systems tend to exhibit low cmc values and form small micellar particles, which is beneficial for structural studies.^[^ ^49^ ^]^ Fluorinated detergents can interfere less with protein–lipid interactions compared to detergents with pure hydrocarbon tails,^[^ ^50^ ^]^ which can potentially help with gaining insights into lipidomes that co‐purify with proteins.^[^ ^5^ ^]^ However, immiscibility between fluorinated detergents and lipid membranes has limited utility for protein purification for years. Recent achievements in the optimization of fluorinated detergents have sufficiently addressed this problem and leave this innovation hurdle behind. Recent fluorinated detergent architectures can readily solubilize proteins from membranes, which paves the way for studying the utility of fluorinated detergents for a better contextualizing of the role of lipid binding in protein structure and function.^[^ ^50^, ^52^ ^]^

Fluorinated amphiphiles, which are able to form nanodiscs without MSPs, can fragment membranes into nanodiscs. Fluorinated detergents are expected to maintain the natural lipid arrangement surrounding proteins better than hydrocarbon detergents. The lipophobic character of fluorinated detergents that surround nanodiscs result in slow, less efficient lipid exchange compared to hydrocarbon‐based systems.^[^ ^55^ ^]^

The ability to maintain membrane protein–lipid interactions during purification is a crucial skill for different structural and functional studies.^[^ ^5^, ^73^ ^]^ Recently developed non‐ionic hybrid detergents are the first demonstration of a technology that can gradually control the relative degree to which membrane proteins co‐purify with lipids. Interestingly, these hybrid detergents^[^ ^26^ ^]^ were obtained from a top‐down design in which mathematical principles were employed to match the conical shapes and HLB values of hybrid detergents to those of protein‐stabilizing detergents.^[^ ^28^, ^33^, ^34^ ^]^ This breakthrough challenges the dogma that the selection and design of detergents in membrane protein purification depends more on empirical factors than on scientific principles.^[^ ^11^, ^13^ ^]^ The combination of non‐ionic hybrid detergents and a carefully designed protein purification pipeline with native mass spectrometry readout led to the identification of non‐canonical glycolipid associations that are relevant for the structure and function of inner membranes in Gram‐negative bacteria and antibiotic research.^[^ ^26^, ^31^ ^]^

In addition to progress in detergent chemistry, it becomes increasingly apparent that detergent exchange strategies can determine experimental successes.^[^ ^24^, ^63^, ^67^ ^]^ Since detergents that enable both the purification of intact proteins and analysis are hard to find, detergents are routinely exchanged between individual steps of purification and/or analysis. The relative degree of detergent exchange is case specific. Future research is required to clarify the underappreciated bias that mixed micellar assemblies from incomplete detergent exchanges can have on membrane protein purification and analysis. Technologies that enable an easy, fast, and accurate quantification of detergent mixtures in membrane protein preparation are still missing but would significantly accelerate our ability to answer the question of how the degree to which detergents are exchanged influences experimental outcomes. In this regard, cleavable detergents can improve detergent exchange processes and enable applications in which detergents cause unattractive bias on measurement data but are mandatory to secure analyte stability in solution, such as in the cases of mass spectrometry applications.^[^ ^72^ ^]^ To reduce time and costs associated with detergent exchange, living detergents have recently been established in which detergents are equipped with functional tags that enable bioorthogonal modifications with externally introduced structural elements between protein purification steps.^[^ ^74^ ^]^ The concept of living detergents increases the diversity of detergentome and can enable new discoveries.

Taken together, the growing chemical diversity in the detergentome delivers exciting opportunities to develop tools that facilitate the purification and biophysical characterization of intact membrane proteins and their complexes with lipids, which will continue in enabling the discovery of novel biological findings.

## 4 Experimental Section

A PubChem‐ and PubMed‐based search for English‐written literature using the search term “novel detergents, membrane protein studies” [Title/Abstract] OR “novel detergents, membrane protein, downstream” OR “surfactant protein purification” [Title/Abstract] AND 2021/01/01 [Date–Publication]: 2024/05/17 [Date–Publication] was performed on 17 May 2024. Furthermore, a PubMed‐based search for English‐written literature using the search term “detergent protein purification” [Title/Abstract] AND 2021/01/01 [Date–Publication]: 2024/05/10 [Date–Publication] was performed on 10 May 2024. Additionally, scientific literature and reference lists of publications within the scope of the current article were mined to identify relevant, but not PubChem‐ or PubMed‐listed publications. Literature related to native mass spectrometry has been selected based on the authors' experience.

## Author Contributions

Literature search was done by K. Alker, J.‐S. Behnke, and L.H. Urner. The manuscript was prepared by K. Alker and L.H. Urner with input from J.‐S. Behnke.

## Conflict of Interests

The authors declare no conflict of interest.

## Data Availability

The data that support the findings of this study are available from the corresponding author upon reasonable request.

## References

[1] M. L. Chiu , Curr. Protoc. Protein Sci. 2012, 29, 29.1.1–29.1.8, 10.1002/0471140864.ps2901s67. 22294326

[2] J. P. Whitelegge , Anal. Chem. 2013, 85, 2558–2568. 23301778 10.1021/ac303064a PMC3664232

[3] R. M. Garavito , S. Ferguson‐Miller , J. Biol. Chem. 2001, 276, 32403–32406. 11432878 10.1074/jbc.R100031200

[4] C. J. Jeffery , Curr. Protoc. Protein Sci. 2016, 83, 29.15.1–29.15.15. 10.1002/0471140864.ps2915s83 26836409

[5] L. H. Urner , Curr. Opin. Chem. Biol. 2022, 69, 102157. 35580377 10.1016/j.cbpa.2022.102157

[6] L. Tiefenauer , S. Demarche , Materials 2012, 5, 2205–2242.

[7] A. J. Miles , B. A. Wallace , Chem. Soc. Rev. 2016, 45, 4859–4872. 27347568 10.1039/c5cs00084j

[8] E. P. Carpenter , K. Beis , A. D. Cameron , S. Iwata , Curr. Opin. Struct. Biol. 2008, 18, 581–586. 18674618 10.1016/j.sbi.2008.07.001 PMC2580798

[9] S. J. Opella , Annu. Rev. Anal. Chem. (Palo Alto Calif) 2013, 6, 305–328. 23577669 10.1146/annurev-anchem-062012-092631 PMC3980955

[10] J. E. Keener , G. Zhang , M. T. Marty , Anal. Chem. 2021, 93, 583–597. 33115234 10.1021/acs.analchem.0c04342 PMC7855921

[11] E. Desuzinges Mandon , M. Agez , R. Pellegrin , S. Igonet , A. Jawhari , Anal. Biochem. 2017, 517, 40–49. 27847172 10.1016/j.ab.2016.11.008

[12] S. Kalipatnapu , A. Chattopadhyay , IUBMB life 2005, 57, 505–512. 16081372 10.1080/15216540500167237

[13] L. H. Urner , I. Liko , H.‐Y. Yen , K.‐K. Hoi , J. R. Bolla , J. Gault , F. G. Almeida , M.‐P. Schweder , D. Shutin , S. Ehrmann , R. Haag , C. V. Robinson , K. Pagel , Nat. Commun. 2020, 11, 564. 31992701 10.1038/s41467-020-14424-8 PMC6987200

[14] A. Helenius , K. Simons , Biochim. Biophys. Acta 1975, 415, 29–79. 1091302 10.1016/0304-4157(75)90016-7

[15] A. M. Seddon , P. Curnow , P. J. Booth , Biochim. Biophys. Acta 2004, 1666, 105–117. 15519311 10.1016/j.bbamem.2004.04.011

[16] C. Tanford , J. A. Reynolds , Biochim. Biophys. Acta 1976, 457, 133–170. 135582 10.1016/0304-4157(76)90009-5

[17] M. Le Maire , P. Champeil , J. V. Moller , Biochim. Biophys. Acta 2000, 1508, 86–111. 11090820 10.1016/s0304-4157(00)00010-1

[18] G. G. Privé , Methods 2007, 41, 388–397. 17367711 10.1016/j.ymeth.2007.01.007

[19] L. H. Urner , I. Liko , K. Pagel , R. Haag , C. V. Robinson , Biochim. Biophys. Acta Biomembr. 2022, 1864, 183958. 35551920 10.1016/j.bbamem.2022.183958

[20] N. T. Johansen , F. G. Tidemand , M. C. Pedersen , L. Arleth , Biochimie 2023, 205, 3–26. 35963461 10.1016/j.biochi.2022.07.014

[21] G. Ratkeviciute , B. F. Cooper , T. J. Knowles , Biochem. Soc. Trans. 2021, 49, 1763–1777. 34415288 10.1042/BST20210181 PMC8421053

[22] B. C. Choy , R. J. Cater , F. Mancia , E. E. Pryor , Biochim. Biophys. Acta Biomembr. 2021, 1863, 183533. 33340490 10.1016/j.bbamem.2020.183533 PMC7856071

[23] C. Le Bon , B. Michon , J.‐L. Popot , M. Zoonens , Q. Rev. Biophys. 2021, 54, e6. 33785082 10.1017/S0033583521000044

[24] J.‐S. Behnke , L. H. Urner , Anal. Bioanal. Chem. 2023, 415, 3897–3909. 36808272 10.1007/s00216-023-04584-z PMC10328889

[25] E. Reading , I. Liko , T. M. Allison , J. L. P. Benesch , A. Laganowsky , C. V. Robinson , Angew. Chem. Int. Ed. Engl. 2015, 54, 4577–4581. 25693501 10.1002/anie.201411622

[26] L. H. Urner , F. Fiorentino , D. Shutin , J. B. Sauer , M. T. Agasid , T. J. El‐Baba , J. R. Bolla , P. J. Stansfeld , C. V. Robinson , J. Am. Chem. Soc. 2024, 146, 11025–11030. 38604609 10.1021/jacs.3c14358 PMC11046432

[27] C. Kirschbaum , K. Greis , S. Gewinner , W. Schöllkopf , G. Meijer , G. von Helden , K. Pagel , L. H. Urner , ChemPlusChem 2024, 89, e202400340. 39031638 10.1002/cplu.202400340

[28] L. H. Urner , A. Ariamajd , A. Weikum , Chem. Sci. 2022, 13, 10299–10307. 36277644 10.1039/d2sc03130b PMC9473536

[29] J. N. Israelachvili , D. J. Mitchell , B. W. Ninham , J. Chem. Soc., Faraday Trans. 2 1976, 72, 1525.

[30] W. C. Griffin , J. Soc. Cosmet. Chem 1949, 311–326.

[31] L. H. Urner , Analytical Science Magazine WILEY, Hoboken, NJ 2024, pp. 27–29.

[32] H. J. Lee , H. S. Lee , T. Youn , B. Byrne , P. S. Chae , Chem. 2022, 8, 980–1013.

[33] L. H. Urner , F. Junge , F. Fiorentino , T. J. El‐Baba , D. Shutin , G. Nölte , R. Haag , C. V. Robinson , Chem. Eur. J. 2023, 29, e202300159. 36897295 10.1002/chem.202300159

[34] J. N. Umbreit , J. L. Strominger , PNAS 1973, 70, 2997–3001. 4200727 10.1073/pnas.70.10.2997 PMC427155

[35] K. H. Cho , P. Hariharan , J. S. Mortensen , Y. Du , A. K. Nielsen , B. Byrne , B. K. Kobilka , C. J. Loland , L. Guan , P. S. Chae , Chembiochem 2016, 17, 2334–2339. 27981750 10.1002/cbic.201600429 PMC5500196

[36] P. S. Chae , A. C. Kruse , K. Gotfryd , R. R. Rana , K. H. Cho , S. G. F. Rasmussen , H. E. Bae , R. Chandra , U. Gether , L. Guan , B. K. Kobilka , C. J. Loland , B. Byrne , S. H. Gellman , Chem. Eur. J. 2013, 19, 15645–15651. 24123610 10.1002/chem.201301423 PMC3947462

[37] A. K. Singh , M. Seewald , B. Schade , C. Zoister , R. Haag , L. H. Urner , Commun. Chem. 2025, 8, 70. 40057629 10.1038/s42004-025-01477-3 PMC11890857

[38] E. Nji , Y. Chatzikyriakidou , M. Landreh , D. Drew , Nat. Commun. 2018, 9, 4253. 30315156 10.1038/s41467-018-06702-3 PMC6185904

[39] S. Lee , A. Mao , S. Bhattacharya , N. Robertson , R. Grisshammer , C. G. Tate , N. Vaidehi , J. Am. Chem. Soc. 2016, 138, 15425–15433. 27792324 10.1021/jacs.6b08742 PMC5148649

[40] M. T. Agasid , L. Sørensen , L. H. Urner , J. Yan , C. V. Robinson , J. Am. Chem. Soc. 2021, 143, 4085–4089. 33711230 10.1021/jacs.0c11837 PMC7995251

[41] H. S. Lee , M. Das , F. Mahler , W. Ahmed , H. Wang , J. S. Mortensen , P. Hariharan , L. Ghani , B. Byrne , L. Guan , C. J. Loland , S. Keller , P. S. Chae , Chem. Asian J. 2022, 17, e202200941. 36253323 10.1002/asia.202200941

[42] M. Ehsan , H. Wang , S. Katsube , C. F. Munk , Y. Du , T. Youn , S. Yoon , B. Byrne , C. J. Loland , L. Guan , B. K. Kobilka , P. S. Chae , Chembiochem 2022, 23, e202200027. 35129249 10.1002/cbic.202200027 PMC8986615

[43] S. Yoon , H. E. Bae , P. Hariharan , A. Nygaard , B. Lan , M. Woubshete , A. Sadaf , X. Liu , C. J. Loland , B. Byrne , L. Guan , P. S. Chae , Bioconjug. Chem. 2024, 35, 223–231. 38215010 10.1021/acs.bioconjchem.3c00507 PMC10970486

[44] L. Ghani , X. Zhang , C. F. Munk , P. Hariharan , B. Lan , H. S. Yun , B. Byrne , L. Guan , C. J. Loland , X. Liu , P. S. Chae , Bioconjug. Chem. 2023, 34, 739–747. 36919927 10.1021/acs.bioconjchem.3c00042 PMC10145683

[45] L. Ghani , S. Kim , M. Ehsan , B. Lan , I. H. Poulsen , C. Dev , S. Katsube , B. Byrne , L. Guan , C. J. Loland , X. Liu , W. Im , P. S. Chae , Chem. Sci. 2023, 14, 13014–13024. 38023530 10.1039/d3sc03543c PMC10664503

[46] L. Ghani , S. Kim , H. Wang , H. S. Lee , J. S. Mortensen , S. Katsube , Y. Du , A. Sadaf , W. Ahmed , B. Byrne , L. Guan , C. J. Loland , B. K. Kobilka , W. Im , P. S. Chae , Chem. Eur. J. 2022, 28, e202200116. 35238091 10.1002/chem.202200116 PMC9007890

[47] M. Ehsan , L. Ghani , B. Lan , S. Katsube , I. H. Poulsen , X. Zhang , M. Arslan , B. Byrne , C. J. Loland , L. Guan , X. Liu , P. S. Chae , Chembiochem 2025, 26, e202400958. 39779472 10.1002/cbic.202400958 PMC11875885

[48] H. J. Lee , M. Ehsan , X. Zhang , S. Katsube , C. F. Munk , H. Wang , W. Ahmed , A. Kumar , B. Byrne , C. J. Loland , L. Guan , X. Liu , P. S. Chae , Chem. Sci. 2022, 13, 5750–5759. 35694361 10.1039/d2sc00539e PMC9116450

[49] M. Yang , Y. Dai , F. Zhou , X. Zhou , Y. Qiu , Y. Tan , S. Zhao , D. Xue , F. Zhao , H. Tao , Chem. Eur. J. 2025, 31, e202404520. 39777805 10.1002/chem.202404520

[50] D. Cornut , M. Soulié , P. Guillet , K. Onyia , F. Mahler , S. Keller , A. Moreno , G. Durand , ChemPlusChem 2025, 90, e202400740. 39812556 10.1002/cplu.202400740

[51] M. Soulié , A. Deletraz , M. Wehbie , F. Mahler , I. Bouchemal , A. Le Roy , I. Petit‐Härtlein , S. Keller , A. Meister , E. Pebay‐Peyroula , C. Breyton , C. Ebel , G. Durand , Biochimie 2023, 205, 40–52. 36375632 10.1016/j.biochi.2022.11.003

[52] M. Soulié , A. Deletraz , M. Wehbie , F. Mahler , B. Chantemargue , I. Bouchemal , A. Le Roy , I. Petit‐Härtlein , F. Fieschi , C. Breyton , C. Ebel , S. Keller , G. Durand , ACS Appl. Mater. Interfaces 2024, 16, 32971–32982. 38885044 10.1021/acsami.4c03359

[53] J. W. Missel , N. Salustros , E. R. Becares , J. H. Steffen , A. G. Laursen , A. S. Garcia , M. M. Garcia‐Alai , Č. Kolar , P. Gourdon , K. Gotfryd , Curr. Res. Struct. Biol. 2021, 3, 85–94. 34235488 10.1016/j.crstbi.2021.03.002 PMC8244287

[54] F. Mangin , V. Chauhan , P. Guillet , M. Damian , M. Soulié , J.‐L. Banères , G. Durand , ACS Appl. Polym. Mater. 2024, 6, 3139–3149.

[55] F. Mahler , A. Meister , C. Vargas , G. Durand , S. Keller , Small 2021, 17, 2103603. 10.1002/smll.202103603 34674382

[56] P. S. Chae , S. G. F. Rasmussen , R. R. Rana , K. Gotfryd , R. Chandra , M. A. Goren , A. C. Kruse , S. Nurva , C. J. Loland , Y. Pierre , D. Drew , J.‐L. Popot , D. Picot , B. G. Fox , L. Guan , U. Gether , B. Byrne , B. Kobilka , S. H. Gellman , Nat. Methods 2010, 7, 1003–1008. 21037590 10.1038/nmeth.1526 PMC3063152

[57] V. Wycisk , M.‐C. Wagner , L. H. Urner , ChemPlusChem 2024, 89, e202300386. 37668309 10.1002/cplu.202300386

[58] F. Zhao , Z. Zhu , L. Xie , F. Luo , H. Wang , Y. Qiu , W. Luo , F. Zhou , D. Xue , Z. Zhang , T. Hua , D. Wu , Z.‐J. Liu , Z. Le , H. Tao , Chem. Eur. J. 2022, 28, e202201388. 35608006 10.1002/chem.202201388

[59] M. Yang , W. Luo , W. Zhang , H. Wang , D. Xue , Y. Wu , S. Zhao , F. Zhao , X. Zheng , H. Tao , Chem. Asian J. 2022, 17, e202200372. 35575910 10.1002/asia.202200372

[60] P. Guillet , F. Mahler , K. Garnier , G. Nyame Mendendy Boussambe , S. Igonet , C. Vargas , C. Ebel , M. Soulié , S. Keller , A. Jawhari , G. Durand , Langmuir 2019, 35, 4287–4295. 30767533 10.1021/acs.langmuir.8b02842

[61] G. N. M. Boussambe , P. Guillet , F. Mahler , A. Marconnet , C. Vargas , D. Cornut , M. Soulié , C. Ebel , A. Le Roy , A. Jawhari , F. Bonneté , S. Keller , G. Durand , Methods 2018, 147, 84–94. 29857192 10.1016/j.ymeth.2018.05.025

[62] J. Baron , L. Bauernhofer , S. R. A. Devenish , S. Fiedler , A. Ilsley , S. Riedl , D. Zweytick , D. Glueck , A. Pessentheiner , G. Durand , S. Keller , Anal. Chem. 2023, 95, 587–593. 36574263 10.1021/acs.analchem.2c03168 PMC9850350

[63] I. Levesque , B. R. Juliano , K. F. Parson , B. T. Ruotolo , J. Am. Soc. Mass Spectrom 2023, 34, 2662–2671. 37956121 10.1021/jasms.3c00230

[64] J. R. Bolla , R. A. Corey , C. Sahin , J. Gault , A. Hummer , J. T. S. Hopper , D. P. Lane , D. Drew , T. M. Allison , P. J. Stansfeld , C. V. Robinson , M. Landreh , Angew. Chem. 2020, 59, 3523–3528. 31886601 10.1002/anie.201914411 PMC7065234

[65] V. Kotov , K. Bartels , K. Veith , I. Josts , U. K. T. Subhramanyam , C. Günther , J. Labahn , T. C. Marlovits , I. Moraes , H. Tidow , C. Löw , M. M. Garcia‐Alai , Sci. Rep. 2019, 9, 10379. 31316088 10.1038/s41598-019-46686-8 PMC6637136

[66] D. Sjöstrand , R. Diamanti , C. A. K. Lundgren , B. Wiseman , M. Högbom , Protein Sci. 2017, 26, 1653–1666. 28543736 10.1002/pro.3196 PMC5521553

[67] V. Chaptal , F. Delolme , A. Kilburg , S. Magnard , C. Montigny , M. Picard , C. Prier , L. Monticelli , O. Bornert , M. Agez , S. Ravaud , C. Orelle , R. Wagner , A. Jawhari , I. Broutin , E. Pebay‐Peyroula , J.‐M. Jault , H. R. Kaback , M. Le Maire , P. Falson , Sci. Rep. 2017, 7, 41751. 28176812 10.1038/srep41751 PMC5297245

[68] T. Kawate , E. Gouaux , Structure 2006, 14, 673–681. 16615909 10.1016/j.str.2006.01.013

[69] Y. Zhu , B.‐J. Peng , S. Kumar , L. Stover , J.‐Y. Chang , J. Lyu , T. Zhang , S. Schrecke , D. Azizov , D. H. Russell , L. Fang , A. Laganowsky , Nat. Commun. 2023, 14, 5676. 37709761 10.1038/s41467-023-41429-w PMC10502129

[70] L. Liu , Z. Zhu , F. Zhou , D. Xue , T. Hu , W. Luo , Y. Qiu , D. Wu , F. Zhao , Z. Le , H. Tao , ACS omega 2021, 6, 21087–21093. 34423216 10.1021/acsomega.1c02894 PMC8375090

[71] K. A. Brown , M. K. Gugger , D. S. Roberts , D. Moreno , P. S. Chae , Y. Ge , S. Jin , Langmuir 2023, 39, 1465–1473. 36638323 10.1021/acs.langmuir.2c02820 PMC10164600

[72] K. A. Brown , M. K. Gugger , Z. Yu , D. Moreno , S. Jin , Y. Ge , Anal. Chem. 2023, 95, 1801. 10.1021/acs.analchem.2c03916 PMC10323036 36608324

[73] D. S. Chorev , C. V. Robinson , Nat. Chem. Biol. 2020, 16, 1285–1292. 33199903 10.1038/s41589-020-0574-1 PMC7116504

[74] Y. Dai , M. Yang , W. Luo , Y. Qiu , F. Zhou , X. Zheng , F. Zhao , X. Yao , S. Zhao , H. Tao , Chem. Eur. J. 2025, 31, e202501128. 40192258 10.1002/chem.202501128

